# From Development to Validation: Exploring the Efficiency of Numetrive, a Computerized Adaptive Assessment of Numerical Reasoning

**DOI:** 10.3390/bs15030268

**Published:** 2025-02-25

**Authors:** Marianna Karagianni, Ioannis Tsaousis

**Affiliations:** 1 Department of Psychology, School of Social Sciences, University of Crete, 74100 Rethymno, Greece; 2 Department of Psychology, National and Kapodistrian University of Athens, 15784 Athens, Greece; ioantsaousis@psych.uoa.gr

**Keywords:** computerized adaptive testing, CAT, Numetrive, numerical reasoning, simulation study, item banking, item equating

## Abstract

The goal of the present study is to describe the methods used to assess the effectiveness and psychometric properties of Numetrive, a newly developed computerized adaptive testing system that measures numerical reasoning. For this purpose, an item bank was developed consisting of 174 items concurrently equated and calibrated using the two-parameter logistic model (2PLM), with item difficulties ranging between −3.4 and 2.7 and discriminations spanning from 0.51 up to 1.6. Numetrive constitutes an algorithmic combination that includes maximum likelihood estimation with fences (MLEF) for *θ* estimation, progressive restricted standard error (PRSE) for item selection and exposure control, and standard error of estimation as the termination rule. The newly developed CAT was evaluated in a Monte Carlo simulation study and was found to perform highly efficiently. The study demonstrated that on average 13.6 items were administered to 5000 simulees while the exposure rates remained significantly low. Additionally, the accuracy in determining the ability scores of the participants was exceptionally high as indicated by various statistical indices, including the bias statistic, mean absolute error (MAE), and root mean square error (RMSE). Finally, a validity study was performed, aimed at evaluating concurrent, convergent, and divergent validity of the newly developed CAT system. Findings verified Numertive’s robustness and applicability in the evaluation of numerical reasoning.

## 1. Introduction

Computerized adaptive testing (CAT) is an efficient form of computer-based assessment that evaluates participants’ latent traits (theta *θ*). CAT systems tailor the characteristics of the items administered and the length of the test to each examinee’s latent trait level. Thus, they substantially reduce the testing time and the number of items used in an assessment while delivering more accurate latent trait evaluations than linear computer based tests (CBTs) and paper and pencil tests (Mao & Xin, 2013). Research about CAT has advanced greatly in the last 20 years and it has become the leading psychometric technique in cognitive and non-cognitive assessments in both educational and psychological settings (Moore et al., 2023). Computerized adaptive tests (CATs), select an item that matches the examinee’s latent trait level from an item bank, and administer said item. The examinee’s *θ* is then updated in accordance with the response to this item and all previous responses. This is an iterative process, and it ceases according to a predetermined stopping rule when the examinee’s *θ* has been accurately estimated (N. E. Thompson & Weiss, 2011). CAT systems are feasible due to their development within the item response theory (IRT) framework, a set of statistical models that establish a relationship between item characteristics and the examinees’ latent traits, by placing them in the same continuum (Embretson & Reise, 2013). IRT is a very powerful statistical technique; it yields comprehensive item analyses, accurate *θ* estimations, and robust equating techniques (Lang & Tay, 2021). CAT is the primary assessment method in many high-stake testing operations worldwide, such as Test of English as a Foreign Language–TOEFL, American Society for Clinical Pathology Board of Certification Examinations, Graduate Management Admission Test–GMAT, and many more.

The development of IRT and computing technology has fundamentally changed modern assessment, with the most important aspect being adaptive testing. CATs display some important advantages compared to linear computer-based tests (CBTs) and paper and pencil tests (PPTs). The most important is improved measurement precision, while the test length of a CAT system is considerably smaller than a linear test. Furthermore, CAT is argued to reduce systematic sources of measurement error such as floor and ceiling effects, and thus increases the assessment process’s validity (Seo, 2017). Other CAT advantages include flexible testing schedules, increased test security, item exposure control, content balancing, easy updating of the item banks, and rapid scoring. Additionally, it is an examinee-friendly test-taking experience due to reduced fatigue that stems from shorter tests (Linacre, 2000). Many examinees could benefit from the advantages offered by CATs, such as students with special education needs (SEN). SEN students are evaluated with fewer items, which is beneficial as students with disabilities may encounter challenges such as maintaining focus, fatigue, etc. (Ebenbeck & Gebhardt, 2024). Additionally, CAT ensures that lower-performing examinees are not overwhelmed by addressing floor effects. For high-performing examinees, CATs present more challenging items, preventing ceiling effects (Weiss & Şahin, 2024). CATs also display some drawbacks like being a fairly complicated technique that requires a sizable amount of both financial and human resources. However, the benefits significantly exceed the drawbacks of CAT assessments (Meijer & Nering, 1999). All those advantages are made possible due to the IRT-based algorithms that lie in the components of a CAT system.

### 1.1. CAT System Structure

A CAT system encompasses an item bank, which comprises a set of items that collectively provide information across the entire spectrum of the latent trait. The assessment begins and an initial item is administered based on a predetermined decision rule. For instance, if no prior information exists about the examinee’s theta, the same item can be administered to all examinees, or an item randomly selected around the *θ* range of 0 (Bjorner et al., 2007; Tsaousis et al., 2021).

Typically, a CAT system comprises three iterative processes: the *θ* estimation, the termination rule, and the item selection. The *θ* estimation process determines the examinees’ latent trait level based on their answers to the selected items. The most popular *θ* estimation methods used in CAT systems are maximum likelihood estimation (MLE), an unbiased method but unable to handle non-mixed response vectors (all correct or incorrect responses); the Bayesian-based methods modal a posteriori (MAP) and expected a posteriori (EAP), which have been found to produce biased estimations due to the use of a prior distribution and weighted likelihood, which can result in more than one *θ* estimation for the same response vector (WL) (Han, 2018a; Magis et al., 2017; Van Der Linden & Pashley, 2010). Han (2016) suggested the maximum likelihood estimation with fences (MLEF) as an alternative method for *θ* estimation. MLEF can handle non-mixed vectors by introducing two hypothetical items with fixed responses that act as “fences” and create a controlled environment where the calculated scores fall within a defined interval, and just like MLE is an unbiased estimation method. This technique essentially creates a controlled environment within which the estimation takes place, ensuring that the calculated scores fall within a defined interval, thereby enhancing the precision and relevance of the score estimation process (Han, 2016). For the 2PL model, the MLEF is calculated as follows:

(1) $$\theta =ln{P(\theta s}_{LB})+\left(1-ln{P(\theta s}_{UB}\right)+\sum_{i=1}^{I}[u_{si}ln{P(\theta }_{si})+\left(1-u_{si}\right)ln(1-{P(\theta }_{si}))]$$

where *P(*${\theta s}_{LB}$*)* and *P*${(\theta s}_{UB}$*)* are the item response functions of the lower and upper fences, respectively, *θs* is the ability level that examinee *s* possesses, *u_si_* is the response of examinee *s* to item *i*, and *P(θ_si_)* is the response function of item *i*.

As soon as the first item is administered and scored, the termination rule criterion is implemented for the first time and then is put into effect every time a new item is administered. The termination rule determines when the system will end the assessment. In fixed-length CATs, where a predetermined number of items is administered to all examinees, the assessment ends once the maximum number of items has been reached. It is a straightforward rule, yet it results in variability in the precision of the *θ* estimation due to the differences in the total item information provided by each examinee’s response matrix (Choi et al., 2010). In variable-length CATs, the stopping rules aim to attain a specific level of measurement precision for all examinees. The most common stopping rule is based on the standard error (SE), where items are administered until the examinees’ *θ* estimate reaches a prespecified level of measurement precision. A reasonable cut-off value to ensure measurement precision is when the standard error drops below 0.35 (Stafford et al., 2018). Another common stopping method is based on the minimum information (MI). The MI rule terminates CATs when no more items in the test bank are capable of providing more than a specified minimal amount of psychometric information at the current *θ* estimate. In the literature, when comparing the two rules, it has been found that the SE stopping rule yields less floor/ceiling effects and delivers shorter tests while providing more accurate measurement precision than the MI (Dodd et al., 1993).

The item selection process encompasses three components: item selection, content balancing, and item exposure control. A wide variety of item selection algorithms exist, but only few are actually implemented in CAT systems. The most well-known item selection method is maximum Fisher information (MFI). It selects and administers the item that maximizes Fisher information at a given latent trait *θ* estimate. It is a straightforward and easy method to implement, but it is rarely used in CAT commercial systems since it excessively uses items with high a parameter that leads to poor item bank utilization (Han, 2018a). Furthermore, MFI depends on the current *θ* of the examinee, and the items it chooses are not necessarily the most informative ones for the examinee’s true *θ*. This leads to compromised measurement precision (Sibley Butterfield, 2016).

To address those issues, Veerkamp and Berger (1997), proposed the interval information (II) and the weighted likelihood information (WLI) criteria. The interval information uses a confidence interval around the true *θ* estimate, weighting each *θ* level in that confidence interval uniformly and values outside confidence interval as 0. The (WLI) gives more weight to the information function when the interim *θ* estimate is closer to the truth *θ*. Both those methods perform slightly better than MFI for extreme *θ* values but overall are not a substantial improvement compared to MFI (S. Chen et al., 2000; Han, 2010; Van Rijn et al., 2002; Veldkamp, 2003). Chang and Ying (1999) proposed the a-Stratified method that addresses the MFI issues by stratifying all α parameter values and reserving items with high α parameter toward the end of the evaluation where the interim *θ* is closer to examinee’s actual *θ*. This method yields stable performance when used with large and optimally designed item banks, and only works with fixed length-CATs.

Han (2012a) also proposed a method that utilizes the α parameters in a more methodical way, called the efficiency balanced information (EBI) criterion. With EBI, items with low α parameter values have a better probability to be selected in the beginning of the CAT evaluation, whereas items with high α values appear more frequently toward the end of the evaluation. Chang and Ying (1996) developed the Kullback–Leibler information criterion, which uses the moving average of the Kullback–Leibler information (KLI) for item selection (Cover & Thomas, 2006). They found that replacing the MFI criterion with the KLI criterion moderately reduced biases and mean squared errors of proficiency estimation in tests with 30 items or less. A different approach to item selection is the difficulty matching criterion, which is employed in Rasch or 1 parameter logistic model (1PL)-based CATs. It selects and administers the item that has the closest b value to the examinee’s current *θ* (Han, 2018a).

When a test has more than one content area, and a specific proportion of items from each content area must be administered, a content balancing procedure must be applied. One commonly used content balancing procedure is proposed by Kingsbury and Zara (1989), where it compares target content proportions from the test specifications to the actual proportions of test administration. The next selected item derives from the content area with the largest deviation between the target and actual proportion. Another method for content sampling is the use of a test script that determines which items are to be administered based on the content area (Han, 2018a).

The last component is item exposure, which refers to limiting the administration of items presenting high discrimination so that they are not used excessively. It is a much-needed procedure in CAT assessment, especially in high stakes tests (Leroux et al., 2013). A widely used method is Kingsbury and Zara’s (1989) Randomesque technique. It selects multiple best items and one of them is randomly administered. The Randomesque technique moderately limits maximum item exposure rates, but it will decrease the overlap in items administered to examinees of similar abilities. Alternative methods, including the Sympson and Hetter (1985) method, the unconditional multinomial method (Stocking & Lewis, 1998), and the conditional multinomial method (Stocking & Lewis, 2000), were also suggested for controlling the overall exposure of the items. However, they are time-consuming because they require simulations to be conducted before CAT administration, during which each item is assigned an exposure parameter (Han, 2018a; Georgiadou et al., 2007).

Han (2009) introduced the fade-away (FA) method, which controls item exposure and can be particularly effective in cloud-based CATs, that is, CATs that are hosted on cloud computing platforms. In order to develop more robust strategies for item exposure, several researchers have developed combined methods that appear to perform better than each strategy alone (Georgiadou et al., 2007). One of the most well-known combined strategies is the progressive restricted strategy (Revuelta & Ponsoda, 1998). The modification, developed for variable-length CATs by McClarty et al. (2006), is called the progressive restricted standard error. The PR-SE combines elements from maximum Fisher information and the restricted maximum information method. Specifically, it brings a random component to maximum information by calculating a weighted value for every administered item as follows:

(2) $$w=\left(1-s\right)R+sI_{i}\left(\theta \right)$$

where

(3) $$s=\frac{0.35}{current\;SE}$$

and *R* is a random number from 0 to H. H is determined as follows. For all items that have not been used, *I_i_(θ)* is calculated with the new *θ* each time, which emerges after the granting and answering of an item. The highest *Ii(θ)* s the H. As the test advances and the SE decreases, the significance of the random factor diminishes (because it is multiplied by 1 *− S*), and more informative items according to Fisher information (displaying high discrimination parameters) are selected. This method was found to outperform the widely used SH and Randomesque methods in both fixed-length and variable-length CATs while administering fewer items, administering almost all of the items from the bank, and reducing the item overlap (Leroux et al., 2013, 2019; Leroux & Dodd, 2016).

### 1.2. Numerical Reasoning

The goal of the present study is to assess the effectiveness and psychometric quality of Numetrive, a newly developed computerized adaptive testing system to measure numerical reasoning in a wide range of contexts including personnel selection, university admission, skill development programs, etc. Numerical reasoning is an important predictor in all these settings for both professional and academic outcomes since it is related to several cognitive skills such as problem-solving, drawing logical conclusions and analyzing quantitative information (e.g., Makransky & Glas, 2013; Dudung et al., 2019). Numerical reasoning is a cognitive ability about the process of handling mathematical properties found in everyday life. It is a set of problem-solving abilities that are used for the interaction with numbers and the mental representations of numerosity. It is the ability to think in numbers rather than the ability to manipulate them (Dudung et al., 2019; Gelman & Gallistel, 1986; Starkey, 1992). It goes beyond simple calculations and involves higher-order mathematical skills. It focuses on determining how to approach and solve problems that contain numerical content. This includes the ability to evaluate a situation, select appropriate problem-solving strategies, draw logical conclusions, develop and describe solutions, and recognize how those solutions can be applied. Numerical reasoning also involves reflecting on solutions to problems to ensure they are logical (Hanson & Dodge, 2018). Numerical reasoning tests consist of simple arithmetic problems of sequences and number representations (Ridwan et al., 2023). Solving a numerical reasoning item usually involves three steps: understanding the problem, devising and carrying out a plan, and looking back (Zhang et al., 2022). They demand high levels of attention, and subjective fatigue increases with the duration of the test, which impacts performance (Ackerman & Kanfer, 2009). In that matter, an examinee-friendly test-taking experience like a CAT system, which reduces fatigue through shorter tests without compromising efficiency (Linacre, 2000), is essential for numerical reasoning.

Numerical reasoning is positively related to other cognitive abilities, such as verbal and abstract reasoning and it has been found to offer added predictive value over deductive reasoning (Hanson & Dodge, 2018). It is widely known and used worldwide as a powerful predictor of overall job performance and training in personnel selection (Salgado, 2017; Salgado & Anderson, 2010). The O*NET database of occupation descriptions, which includes over 900 occupations, describes that numerical reasoning plays a significant role in more than 30% of jobs (O*NET Center, n.d.). Moreover, previous research has shown that numerical reasoning is connected with reduced errors while multitasking (Bühner et al., 2006) and positively related to managerial competency (Perkins & Corr, 2005), innovation, adaptability and creativity, skills that are crucial in navigating though the complexities of technological advancements and the evolving job market in the modern economy (Allworth & Hesketh, 1999; Brynjolfsson & McAfee, 2014; Furnham & Nederstrom, 2010; World Economic Forum, 2020). Additionally, numerical reasoning skills are important in many other areas, such as financial literacy (Darriet et al., 2022) and academic success (Dudung et al., 2019). Its importance is expected to be increased even further in the future as the use of data increases in many jobs. In the modern world of big data and dashboards, numerical reasoning is becoming a crucial reasoning skill (Hanson & Dodge, 2018).

The algorithmic combination that constitutes Numetrive, namely, maximum likelihood estimation with fences (MLEF) for *θ* estimation, progressive restricted standard error (PRSE) for item selection and exposure, and standard error as the termination rule, at least to our knowledge, has not been used before. Each algorithm within this framework was chosen based on evidence from the literature demonstrating its effectiveness in relevant contexts. The resulting CAT system leverages the empirically supported strengths of each algorithm, leading to high performance and efficacy. All three algorithms align with the study’s broader goal of estimating participants’ numerical reasoning ability with high precision, providing insights into performance across the entire *θ* range. Moreover, these algorithms were found to outperform alternative methods, further supporting the robustness of Numetrive (Dodd et al., 1993; Han, 2016; Leroux et al., 2013, 2019; Leroux & Dodd, 2016).

## 2. Materials and Methods

### 2.1. Item Generation and Item Bank Development

The first aim of the study was to develop items measuring numerical reasoning. A subject matter expert (SME) was assembled to generate an initial pool of 220 items of tiered difficulty. These 220 items assess pattern recognition in the form of number sequences and number grids. In number sequences, examinees recognize relationships between numbers (such as arithmetic or geometric progressions), while in number grids (like Sudoku or other puzzles) they identify patterns in how numbers are arranged within the grid (predict or complete missing numbers) (Gelman & Gallistel, 1986; Ridwan et al., 2023). These items involve pattern recognition, internalized number relationships, and efficient numerical processing, which are essential aspects of numerical reasoning (Dudung et al., 2019; Starkey, 1992). The administration of the whole set of 220 items to evaluate their psychometric quality (i.e., item calibration) was not applicable due to the participants’ fatigue and time constraints. To overcome this issue, the items were divided into multiple versions, each containing some common (anchor) items. Common items were essential for equating the different test versions and ensuring all items aligned to a common metric (Muraki et al., 2000). To select the common items, a baseline test version was created, comprising 42 items (12 easy, 20 medium, and 10 difficult). Then, this baseline version was administered to 245 examinees, and the items with the best psychometric characteristics were selected to form the set of common items. Finally, these items were shared into six different versions, each comprising 40 items (30 unique and 10 common items). Then, all versions were combined into a sparse matrix representing the non-equivalent anchor test (NEAT) design and were concurrently calibrated under the 2PL model (Kolen & Brennan, 2014; W. C. Lee & Ban, 2010).

### 2.2. Participants

Several samples were assembled during the developmental phase of Numetrive. The study includes a community sample, encompassing individuals aged 17 to 69 years. First, data were collected from seven different samples—a total of 1723 participants—(*N*1 = 245, *N*2 = 241, *N*3 = 230, *N*4 = 239, *N*5 = 236, *N*6 = 247, *N*7 = 285) for pretesting the items of the item bank. Of them, 751 (43.6%) were male, 746 (43.3%) were female, and 226 (13.1%) did not provide their gender. Regarding their educational background, 743 (43.1%) participants had a high school diploma or lower, 876 (50.8%) had a college degree or higher, and 104 (6.0%) did not provide their educational background. Regarding occupational status, 694 (40.3%) participants identified as white collar, 677 (39.3%) identified as blue collar, 160 (9.3%) were university students, 90 (5.2%) were unemployed, and 102 (5.9%) did not provide their status. Finally, the mean age was 33.89 years (SD = 10.82), and 63 (3.7%) participants did not report their age. This sampling framework ensured that Numetrive would be appropriate for a diverse range of assessment contexts, including educational, organizational, and training. It should be noted that the testing procedure was proctored.

Additionally, an eighth sample was used to perform Numetrive’s validity study. To determine the appropriate sample size, a power analysis was conducted. The analysis was based on two conditions—expected correlation coefficients were set at 0.6 and 0.7 degrees of association—representing large effect sizes. These effect sizes were chosen since concurrent and convergent validity yield medium to large effects. The level of significance (α) was set to 0.01 to minimize the risk of type I error, and α power level to 0.99, aiming for a 99% probability of correctly detecting a true correlation. The results indicated that to detect a correlation of *r* = 0.6, a minimum sample size of 48 participants would be required, and for *r* = 0.7, 32 participants would suffice. The analysis was performed in G*Power (Faul et al., 2007). Data were collected from a sample of 65 participants. Of them, 28 (43.1%) were male, and 32 (49.2%) were female. Furthermore, 18 (27.7%) participants had a high school or lower diploma, and 59 (64.7%) had a college degree or higher. The mean age was 32.35 years (SD = 10.41) and ranged between 19 and 60 years. Participants originated from Greece, and the testing procedure was proctored.

### 2.3. Measures

The ASEP Numerical Reasoning Test (Supreme Council for Civil Personnel Selection, 2022). This IRT-based test comprises 39 items assessing numerical reasoning. All items are in a multiple-choice format and scored as correct (1) or wrong (0). The marginal reliability was found to be 0.90, and the test–retest was 0.87. Regarding validity, a study was conducted to assess concurrent, convergent, and divergent validity. The results provided robust evidence of concurrent validity, which strongly correlated with scores from similar constructs. Furthermore, convergent validity was demonstrated through significant moderate correlations with abstract reasoning. Finally, divergent validity was established, and low correlations were observed between the ASEP Numerical Reasoning Test and unrelated constructs (Supreme Council for Civil Personnel Selection, 2022).

Mentor Abstract Reasoning Test (Hellenic National General Staff, 2017). The Mentor is an abstract reasoning test designed to evaluate an individual’s ability to discern relationships between shapes. The test includes two subscales: progressive and associative matrices, each comprising 20 items. All items are in a multiple-choice format and scored as either correct (1) or wrong (0). The test–retest reliability of the associative matrices subscale is 0.76, and that of the progressive matrices is 0.72. A validity study was conducted and provided robust evidence of concurrent, convergent, and divergent validity (Hellenic National General Staff, 2017).

Penn State Worry Questionnaire—PSWQ (Meyer et al., 1990). PSWQ is a self-report scale consisting of 16 items. It was developed to measure the trait of worry and participants rate each item on a 5-point Likert scale ranging from 1 (“not at all typical of me”) to 5 (“very typical of me”), with higher scores indicating greater levels of worry. Internal reliability is obtained through Cronbach’s alpha, which is 0.93, and demonstrates strong test–retest reliability. This measure has been validated across various groups, showing consistent results in distinguishing between individuals with generalized anxiety disorder and other conditions. The scale has been adapted to the Greek language by Lialou et al. (2020).

Satisfaction with Life Scale—SWLS (Diener et al., 1985) is a self-report scale developed to assess perception of life satisfaction. Participants rate each of the five items on a 7-point Likert scale, ranging from 1 (“strongly disagree”) to 7 (“strongly agree”), with higher scores indicating greater life satisfaction. The SWLS shows high internal consistency (α = 0.87) and strong test–retest reliability (*r* = 0.82). Several studies have demonstrated SWLS’s robust validity. It shows construct validity by correlating positively with subjective well-being measures and produces low correlations with unrelated constructs. The scale has been adapted to the Greek language by Galanakis et al. (2017).

### 2.4. Analytical Strategy

To calibrate the item bank, first, the basic assumptions for IRT were examined (i.e., unidimensionality, local dependency, and monotonicity). To examine the unidimensionality assumption, confirmatory factor analysis (CFA) was performed. The robust weighted least squares (WLSMV) estimator was used since it does not assume normally distributed data and is more appropriate for modeling categorical or ordered data (Brown, 2015). To evaluate model fit, the following goodness-of-fit indices were used: (a) the root mean square error of approximation (RMSEA), (b) the standardized root mean square residual (SRMR), (c) the comparative fit index (CFI), and the Tucker–Lewis index (TLI). CFI and TLI values greater than 0.90 indicate an acceptable fit. Additionally, RMSEA and SRMR values up to 0.08 indicate a reasonable fit to the data, while values less than 0.05 indicate an excellent fit (Hu & Bentler, 2009). Analyses were performed using Mplus 8.1 (Muthen & Muthen, 2019). To evaluate the local dependency assumption, the standardized local dependence statistic (LD–X^2^) (W.-H. Chen & Thissen, 1997) was used. Items exhibiting values greater than 10 were removed to ensure that the items were not overly related to each other. Next, the assumption of item monotonicity was evaluated via visual inspection of the item response functions (IRFs) (Hardouin et al., 2011).

Next, an IRT model selection for the baseline test was performed. Three different models (1PL, 2PL, and 3PL) were examined to determine which one provides the best model fit. Selecting an appropriate IRT model is an essential procedure to ensure the accuracy of parameter estimation, (Terry et al., 2023). Selection of the optimal fitting model was based on the following test-level model-fit indices: −2log-likelihood (22LL; Spiegelhalter et al., 2002), Akaike’s information criterion (AIC; Akaike, 1974), and Bayesian information criterion (BIC; Schwarz, 1978). The model exhibiting the smaller values is considered the most appropriate.

Next, item calibration of the baseline version of Numetrive was performed. First, the item discrimination (a) and item difficulty (b) parameters were evaluated. Discrimination values lower than 0.5 and items with difficulty level beyond and above the *θ* range of −3.5 to 3.5 were removed (Embretson & Reise, 2013). Moreover, the fit of each item to the model’s expectations was evaluated using the S-X^2^ statistic (Orlando & Thissen, 2003). A non-significant S-X^2^ value indicates that the model adequately fits an item. Considering that large samples and multiple comparisons are prone to yield significant X^2^, the Benjamini and Hochberg (1995), correction was applied to adjust the *p*-value. Finally, a differential item functioning (DIF) analysis was performed to detect systematic error caused by group bias, namely gender and educational level (Crane et al., 2006). From this analysis, the common items to be used in the equating procedure were selected based on two criteria: quantity and psychometric properties. Many common items reduce random error (Budescu, 1985; G. Lee & Fitzpatrick, 2008), with a minimum of 20% being in a 40-item test. These items should reflect the difficulty range of the original test, though studies show that even when that is not met, stable results are yielded (Liu et al., 2011). Also important in selecting common items is the discrimination parameter. High-discriminative items lead to more reliable equating (Yu & Osborn-Popp, 2005). All analyses were performed using IRTPRO software, version 4.2 (Cai et al., 2011).

Afterward, the concurrent calibration for equating item parameters across all test forms was performed. Equating methods in CAT systems are used to adjust the difficulty parameter of the items of different test forms so that all items are in a common metric. According to Kolen and Brennan (2014), the concurrent calibration method outperforms alternative methods (e.g., all the conversion methods). The equating process was performed using Xcalibre 4.2 software (Guyer & Thompson, 2014).

To evaluate the effectiveness and the psychometric quality of Numetrive, a Monte Carlo simulation study was conducted. The Monte Carlo simulation method is particularly important in evaluating CAT systems since it allows for mimicking the CAT system’s performance prior to its functional operation (Han, 2018b). Several different indices were used. The first index was the BIAS statistic, an index of systematic measurement error and used to evaluate the measurement precision of the CAT system. It represents the difference between the estimated and true *θ* across all simulees:

(4) $$Bias=\frac{\sum_{i=1}^{1}\left(\hat{\theta_{i}}-\theta_{i}\right)}{Ι}$$

where I is the number of simulees. Additionally, the conditional BIAS (CBIAS) index was estimated, which represents the bias level across the *θ* range (e.g., −2 ≤ *θ* ≤ 2). Previous research has demonstrated that CBIAS is more robust when evaluating CATs (Han, 2018b).

Next, the mean absolute error (MAE) index, a statistic for overall systematic and non-systematic error, and the RMSE index, as another statistic of measurement precision, were assessed as follows:

(5) $$MAE=\frac{\sum_{i=1}^{I}\left|\hat{\theta_{i-}}\theta_{i}\right|}{I}$$

(6) $$RMSE=\sqrt{\frac{{\sum_{i=1}^{I}\hat{(\theta i}-\theta i)}^{2}}{I}}$$

The conditional MAE (CMAE) and the conditional RMSE (CRMSE) for each *θ* level were also computed. Finally, the standard error of estimate (SEE) at the final *θ*, which is $1\sqrt{TIF}$, where TIF is the test information function for the particular test that each simulee completed, was computed:

(7) $$SEE=\frac{1}{\sqrt{TIF}}$$

Next, item exposure statistics were computed to evaluate the system’s security performance. All analyses were conducted using SimulCAT 1.2 software (Han, 2012b).

Finally, to evaluate the validity of the Numetrive, the CAT system was developed by a software engineer and administered along with a series of instruments to evaluate several types of validity, including concurrent, convergent, and divergent validity. To examine the concurrent validity of the Numetrive, an existing test measuring numerical reasoning was used. Convergent validity was evaluated via the administration of an abstract reasoning test. Numerical and abstract reasoning both evaluate aspects of general cognitive ability, and previous research has demonstrated that they share some overlapping cognitive processes (Primi et al., 2010). Finally, divergent validity was assessed by examining the relationship between the Numetrive *θ* estimation and anxiety and life satisfaction. Existing research has not established a link between numerical reasoning and these two constructs, so little to no correlation is expected.

## 3. Results

Initially, the unidimensionality of the baseline version was examined via CFA. The results showed that the one-factor model fits the data adequately, providing support for the unidimensionality assumption [χ^2^ (815) = 964.67, *p* = 0.001; CFI = 0.937, TLI = 0.933, RMSEA = 0.027 (CIs 90% 0.020–0.034), SRMR = 0.117]. Next, the estimation procedures for the Rasch, 2PL, and 3PL models were evaluated to determine the model that provides the best fit. This approach assures the accuracy of item parameter estimation (Terry et al., 2023). Selection of the optimal fitting model was based on the following model-fit indices: −2LL (Spiegelhalter et al., 2002), AIC (Akaike, 1974), and BIC (Schwarz, 1978). Smaller values in all those indices denote a more parsimonious model (Table 1). The results showed that the 2PL IRT model is the model that best fits the data.

Next, item calibration of the baseline version was conducted in order to select the common items for equating the different test versions of the item bank. A total of 16 (out of 42) items were removed due to exhibiting either, local dependency, non-monotonic function, poor item discrimination, item difficulty levels, and item fit. Additionally, none of the items exhibited DIF across both gender and educational level. Then, 10 items representing different difficulty levels across the *θ*, and high discrimination values were selected to comprise the anchor items. Following that, all the combined data sets, including the baseline version, were concurrently calibrated using the 2PL model. The final item bank consisted of 174 items whose item difficulty ranged between −3.4 and 2.7 and discrimination from 0.51 to 1.6. Items were removed if they were displaying low discrimination (<0.5), item difficulty outside the range of −3.5 to 3.5, large SE value for each item parameter, or IRT standardized (z) residuals (after the Benjamini and Hochberg correction was applied) (Benjamini & Hochberg, 1995).

Next, the efficiency of Numetrive was evaluated by a Monte Carlo simulation method. Initially, 5000 response vectors were produced and the corresponding true ability scores (*θ*) were calculated. Following this, the 5000 response vectors were processed within the CAT framework using the 174 items from the Numetrive item bank.

**Figure 1:**
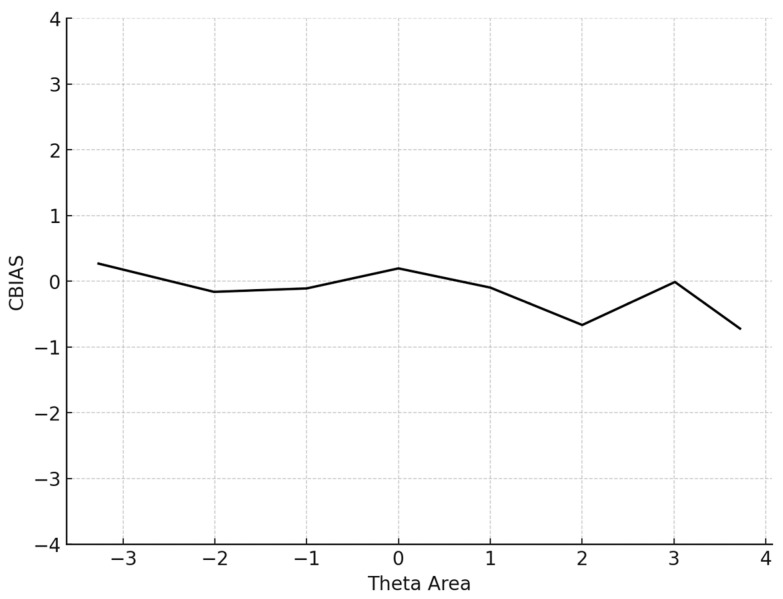
Conditional BIAS (average BIAS in each theta area).

**Figure 2:**
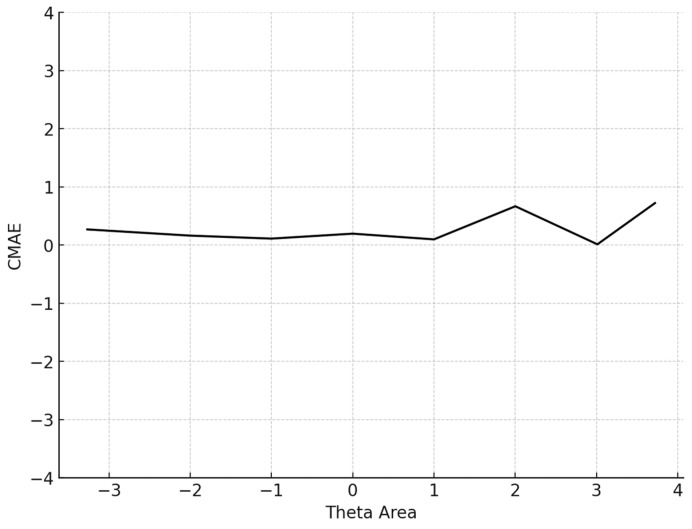
Conditional MAE (average MAE in each theta area). Note: CMAE = conditional mean absolute error.

**Figure 3:**
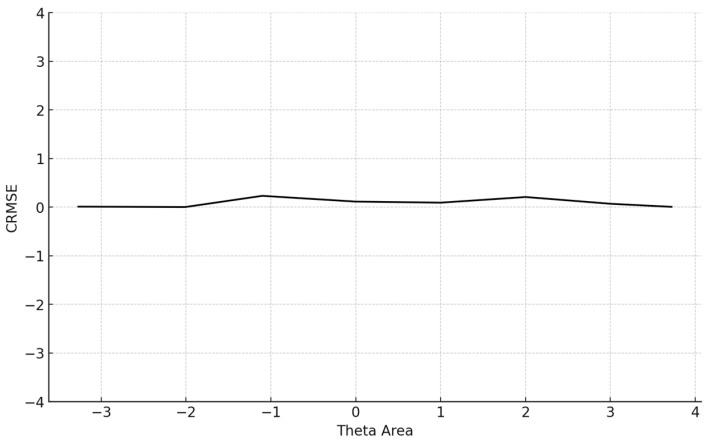
Conditional CRMSE (average RMSE in each theta area). Note: CRMSE = conditional root mean square error.

**Figure 4:**
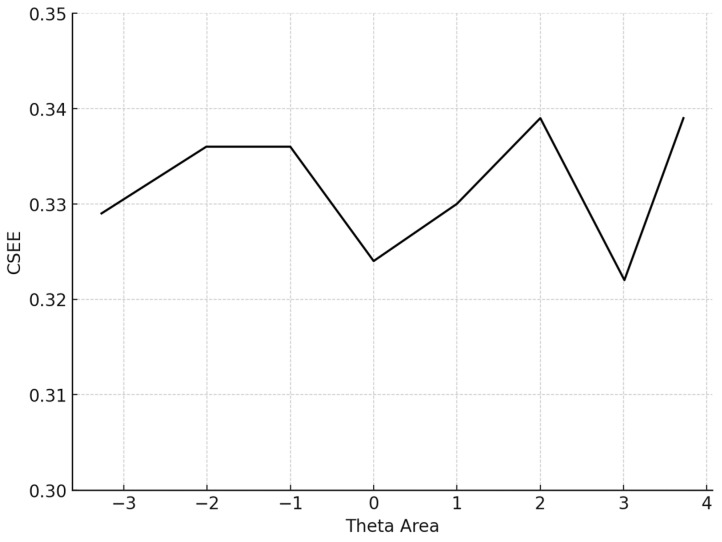
Conditional SEE (average SEE in each theta area). Note: SEE: standard error estimate.

The average number of items administered for the 5000 participants was 13.6 (SD = 14.4). The mean BIAS, an index reflecting the degree to which the CAT system accurately retrieved the true theta parameters by averaging the difference between the estimated and true *θ* across all simulees, was 0.068, signifying proximity to zero. The mean MAE and mean RMSE, both measures of overall systematic and non-systematic errors and precision in measurement, were also very low. Specifically, the MAE was 0.266 and the RMSE is 0.315, indicating high accuracy and precision. The mean item exposure was 8%, indicating that the CAT system effectively prevented item overexposure. Additionally, the standard deviation was notably low (SD = 0.101), evidencing the efficiency of the Numetrive CAT system in preventing excessive use of the items and effectively managing the overall item pool. The findings are presented graphically (Figure 1, Figure 2, Figure 3 and Figure 4).

The study concludes with the psychometric evaluation of the newly developed CAT. First, the Numetrive CAT system demonstrated valid concurrent validity since it correlates highly with an external criterion (i.e., ASEP Numerical Reasoning Test) measuring numerical reasoning (*r* = 0.75, *p* = 0.001). Numetrive also displayed evidence of convergent validity, supported by a moderate positive correlation with an abstract reasoning test (i.e., Mentor Abstract Reasoning Test) (*r* = 0.62, *p* = 0.001). The new CAT system ultimately demonstrated divergent validity since it exhibited non-significant correlations with theoretically unrelated constructs such as anxiety (*r* = 0.17, *p* = 0.16) measured by PSWQ, and life satisfaction (*r* = 0.02, *p =* 0.9) measured by SWLS.

## 4. Discussion

This study aimed at evaluating the effectiveness and psychometric quality of Numetrive, a computerized adaptive testing system designed to assess numerical reasoning for potential use in a wide range of contexts, including personnel selection, university admission, skill development programs, etc. In all these settings, numerical reasoning tests are used for making decisions because they are important predictors of cognitive skills such as problem-solving, drawing logical conclusions, and analyzing quantitative information. All these competencies are essential for success in both academic and professional environments (Dudung et al., 2019; Makransky & Glas, 2013). Numerical reasoning is a critical cognitive skill that involves higher-order problem-solving, such as strategy evaluation and selection, and is essential for tasks encountered in everyday life (Hanson & Dodge, 2018). It has been found to be positively related with other cognitive skills like verbal and abstract reasoning (Primi et al., 2010) and is of widespread importance across various domains. It is a strong predictor of job performance and training (Salgado, 2017; Salgado & Anderson, 2010), and supports innovation, adaptability, and creativity soft-skills that hold an important role in the modern economy (Allworth & Hesketh, 1999; Brynjolfsson & McAfee, 2014; Furnham & Nederstrom, 2010; World Economic Forum, 2020), and plays a key role in financial literacy (Darriet et al., 2022) and academic success (Dudung et al., 2019).

Initially, an item bank was developed comprising 174 items. According to simulation studies, 150 items are enough to yield accurate estimations (Wagner-Menghin & Masters, 2013). Next, the item bank was used to evaluate the effectiveness of the selected combination of Numetrive’s algorithms. Numetrive is a unique combination of algorithms specifically selected to ensure measurement accuracy, low item exposure, and short time administration. For estimating *θ*, the maximum likelihood estimation with fences (MLEF) algorithm was preferred since it is capable of handling non-mixed vectors and provides unbiased estimates of examinee *θ* traits (Han, 2016). Previous findings suggest that MLEF outperforms existing *θ* estimating methods used in CATs. Particularly MLE, although an unbiased method, cannot handle all correct or incorrect responses. The Bayesian methods MAP and EAP have been found to produce biased estimations due to the use of a prior distribution, and WL can result in more than one *θ* estimation for the same response vector (Han, 2018a; Magis et al., 2017; Van Der Linden & Pashley, 2010).

Regarding item selection and exposure algorithms, Numetrive adopts the integrated robust method of progressive restricted standard error. Previous research has found that it outperforms the popular SH and Randomesque methods in both fixed and variable-length CATs, administering fewer items, ensuring broad coverage of the item bank, and reducing item overlap (McClarty et al., 2006; Leroux et al., 2013, 2019; Leroux & Dodd, 2016). Finally, the standard error method was used as a stopping rule since it has been found that it controls floor/ceiling effects effectively and delivers short tests while ensuring accurate measurement precision (e.g., Dodd et al., 1993). Numetrive combines and leverages the established strengths of these algorithms, and it was found to perform highly effectively. According to the results from the simulation study, the accuracy in estimating 5000 simulated examinees’ ability score was very high, as evidenced by several statistical indices and graphs of measurement precision and detection of error, (i.e., CBIAS, CMAE, CRMSE).

This study also demonstrates that Numetrive is very effective in administering a small-scale number of items while maintaining highly accurate ability estimates. On average, only 13.6 items (SD = 14.4) were administered to 5000 simulated examinees. This feature highlights Numetrive’s ability to provide quick, reliable assessments supporting informed confident decisions wherever the test is used (e.g., in personnel selection, university admissions). Additionally, the shorter test length reduces examinee fatigue, ensuring a positive testing experience (Linacre, 2000). Another advantage stemming from shorter tests is that resources like time, effort, and money are saved (Makransky & Glas, 2013). Finally, CAT systems like Numetrive offer the flexibity of unproctored Internet testing (UIT), enabling organizations to administer tests efficiently and safely without requiring physical presence, further saving time, recruitment costs, and other resources (Kantrowitz et al., 2011).

Another notable finding that emerges from the specific algorithm used in Numetrive (PR-SE) is that the majority of the items had an extremely low exposure rate, less than 8%. This minimal exposure ensures substantial item bank security, along with item diversity in the generated item sets. In CAT systems, compromised security resulting from overexposed items could cause threats to both the validity and fairness of the assessment. That is, because examinees who become familiar with the items may respond differently leading to biased results (Han, 2018b). This feature enhances Numetrive’s functionality in environments where fairness along with accuracy are important, like personnel selection and other high stakes environments.

Finally, the present study evaluated the validity of Numetrive by examining its concurrent, convergent, and divergent validity. The results provide robust evidence supporting Numetrive as a valid assessment of numerical reasoning. Particularly, concurrent validity indicated that Numetrive effectively measures numerical reasoning in a way that is consistent with scores of established tests measuring the same construct. This finding suggests that it could serve as an alternative option for numerical reasoning assessment, especially in contexts that could benefit from adaptive testing.

Convergent validity demonstrated that Numetrive shares some overlapping cognitive processes with abstract reasoning, as expected since both are considered facets of general intelligence (g) (Sternberg, 2020). This finding supports previous research which shows that a positive relationship exists between numerical and abstract reasoning (Primi et al., 2010). Furthermore, it supports the existing literature which has shown that reasoning skills such as numerical and abstract are related and contribute to the well-established statistical identity of g (Kazali et al., 2024). Finally, divergent validity provided evidence of the specificity of Numetrive as an assessment focused on numerical reasoning without interference from unrelated psychological variables. This outcome is important because it ensures that Numetrive is measuring the intended construct, and thus confirming its suitability to be used in environments that require targeted evaluation of numerical reasoning skills.

Despite the valuable insights gained, this study has some limitations that should be considered in interpreting the findings. The first arises from the fact that the range of the difficulty parameters in the item bank reaches its maximum value at 2.7 logits. This suggests that to distinguish high-ability individuals accurately, Numetrive needs to administer more items than its average number of 13.6. On the other hand, IRT theta values are generally modeled on a standardized scale ranging approximately between −3 and 3. This range is used because it captures the majority of observed ability levels (N. A. Thompson, 2009). Ability estimates cluster around the central range, rarely exceeding the 2.7 logit. Future research could evaluate the effectiveness of Numetrive’s algorithm combination using an ideal item bank (e.g., evenly distributed item difficulties across −3.5 to 3.5 logits) in a Monte Carlo simulation study to evaluate its full potential. Furthermore, it could compare its effectiveness with some commonly used algorithm combinations in CAT systems. Another limitation of this study is that it does not include an evaluation of Numetrive’s stability over time. This information would confirm that Numetrive measures numerical reasoning in a consistent manner (i.e., when the same examinees are assessed at different time points or at different occasions).

In conclusion, we assessed in this study the effectiveness and psychometric properties of Numetrive, a newly developed computerized adaptive testing system that evaluates numerical reasoning, both with actual examinees as well as in a Monte Carlo real-item simulation. The newly developed CAT was found to perform very efficiently. On average, it administered 13.6 items while the exposure rates remained significantly low. Additionally, the accuracy in determining the ability scores of the participants was exceptionally high. Lastly, a validity study verified its robustness and applicability in evaluating numerical reasoning.

## Figures and Tables

**Table 1:** IRT model selection for the baseline version.

Model	−2LL	AIC	BIC
Rasch	10,632	10,632	10,863
2PL	10,189	10,357	10,651
3PL	10,228	10,480	10,921

Note: AIC = Akaike’s information criterion; BIC = Bayesian information criterion; −2LL = −2log-likelihood.

## Data Availability

Data are available upon request.
